# *Timmia sanjiangyuanensis* (Timmiaceae, Bryophyta), a new species from the Qinghai Plateau, China

**DOI:** 10.3897/phytokeys.276.176402

**Published:** 2026-06-10

**Authors:** Xin-Yin Ma, Jia-Yi Zheng, Xin-Rui Xia, Xiao-Tong Song, Ya-Kun Shi, Yu-Huan Wu, Wen-Zhuan Huang

**Affiliations:** 1 College of Life and Environmental Sciences, Hangzhou Normal University, Hangzhou 311121, China College of Life and Environmental Sciences, Hangzhou Normal University Hangzhou China; 2 Zhejiang Provincial Key Laboratory of Wetland Intelligent Monitoring and Ecological Restoration, Hangzhou Normal University, Hangzhou 311121, China Zhejiang Provincial Key Laboratory of Wetland Intelligent Monitoring and Ecological Restoration, Hangzhou Normal University Hangzhou China; 3 School of Biotechnology, Jiangnan University, No. 1800, Lihu Avenue, Wuxi 214122, China School of Biotechnology, Jiangnan University Wuxi China; 4 Northwest Survey and Planning Institute of the National Forestry and Grassland Administration, Xi’an 710048, China Northwest Survey and Planning Institute of the National Forestry and Grassland Administration Xi’an China

**Keywords:** Mosses, new section, phylogeny, SEM, taxonomy

## Abstract

*Timmia* is a small moss genus with only eight currently accepted species. However, the boundaries and taxonomic status of some taxa remain uncertain and controversial, which has significantly hindered studies on its species diversity. During our 2025 expeditions to the Sanjiangyuan Region in Qinghai Province, China, we discovered a moss resembling *T. sphaerocarpa* with globose capsules, distinguishable by several morphological characters: stomata confined to the capsule base; vegetative and perichaetial leaves with a distinctly differentiated sheathing base; limb laminal cells with distinct mamillae; naked antheridia; sinuose exothecial cells; and spores 2–3 times larger than those of *T. sphaerocarpa*. Morphological and molecular phylogenetic analyses, based on two chloroplast markers (*atp*B-*rbc*L spacer and *trn*L-*trn*F) and the nuclear 26S region, suggest that this moss represents a new species, herein described as *T. sanjiangyuanensis*. A new section, sect. *Pseudosphaerocarpa*, is established to accommodate this species. Descriptions, illustrations and SEM photographs of the peristome and spores of this new species, along with an updated key to *Timmia* species in China, are provided.

## Introduction

As the largest and highest plateau in the world, the Qinghai-Tibet Plateau (QTP) harbours exceptional plant diversity and is recognised as a global biodiversity hotspot (Mittermeier et al. 2004; Orme et al. 2005; Cai et al. 2019). With over 12,000 recorded species of seed plants (Yu et al. 2020), the QTP has long been a focus for botanical research. However, bryophyte diversity in Qinghai Province has been comparatively neglected. Despite encompassing a vast area of approximately 722,300 km^2^, Qinghai has the lowest recorded bryophyte diversity amongst all Chinese provinces, with only about 150 species reported (Qian and Chen 2016; Song et al. 2021). This low number obviously represents a significant underestimate, as evidenced by the recent discovery of several new national records in the region, including *Bryum austriacum* Köckinger, Holyoak & Suanjak (Liu et al. 2022), *Dicranum dispersum* Engelmark (Huang et al. 2024), *D. spadiceum* J.E.Zetterst. (Huang et al. 2024) and *Meesia minor* Brid. (Xia et al. 2025). However, as previous studies primarily focused on the north-western and southern parts of the Qinghai Plateau (Tan and Jia 1997; Wu and Gao 2007), our knowledge of bryophyte diversity in the Qinghai Plateau is still far from fully known, as many areas remain unexplored.

To enhance our understanding of bryophyte diversity in the Qinghai Plateau, we recently conducted a survey focusing on this region (Huang et al. 2024; Xia et al. 2025). In southeast Qinghai Plateau, we discovered an unusual species with globose capsules resembling those of Bartramiaceae (Frey and Stech 2009), but its gametophyte characteristics unequivocally place it within the genus *Timmia* Hedw., characterised by strongly mamillose laminal cells in the limb portion (Brassard 1979, 2007; Frey and Stech 2009). This unique combination of traits suggests that this moss is referable to the Chinese endemic species, *T. sphaerocarpa* Y.Jia & Yang Liu bis. (Jia and Liu 2006). However, several distinguishing features, such as stomata confined to the capsule base, vegetative and perichaetial leaves with a distinctly differentiated sheathing base, limb laminal cells with distinct mamillae, naked antheridia, sinuose exothecial cells and larger spores (36–46 μm in diameter), clearly differentiate this moss from *T. sphaerocarpa* (Jia and Liu 2006). This unique combination of morphological characters captured our attention and prompted our subsequent investigation.

Timmiaceae is a monogeneric family comprising eight currently accepted species (Brinda and Atwood 2025). All species are distributed across the Northern Hemisphere in arctic-montane regions, with only *Timmia megapolitana* Hedw. occurring in temperate zones (Budke and Goffinet 2006). After Brassard’s (1979, 1980, 1984) comprehensive morphological revisions of the genus, molecular studies were conducted by Budke and Goffinet (2006), Hedenäs (2011, 2021) and Belkina et al. (2024). Notably, Brassard (1979) divided the genus into three sections, based on morphological characteristics: sect. *Timmia*, sect. *Timmiaurea* Brassard and sect. *Norvegica* Brassard. This classification was followed by Budke and Goffinet (2006) and Hedenäs (2011). Jia and Liu (2006) added a fourth section, sect. *Sphaerocarpa* Y.Jia & Yang Liu bis, to accommodate their newly-described species *T. sphaerocarpa* Y.Jia & Yang Liu bis. However, a recent study synonymised sect. *Timmiaurea* and sect. *Sphaerocarpa* under sect. *Timmia*, reducing the genus to two sections (Hedenäs 2021). *Timmia* sect. *Norvegica* is characterised by hyaline and fragile cells at the leaf insertion, with leaves readily breaking away from the stem, whereas sect. *Timmia* lacks these features (Brassard 1979, 2007). Based on these morphological characteristic, we hypothesise that the *Timmia* species collected from southeast Qinghai Province belong to sect. *Timmia*.

The objectives of this study are: (1) to confirm the taxonomic status of the unknown moss from Qinghai Province and describe it as a new species, based on morphological and phylogenetic evidence; (2) to test the hypothesis that this moss belongs to sect. *Timmia* using molecular data and (3) to provide an updated key to *Timmia* species in China.

## Materials and methods

### Taxon sampling

The infrageneric relationships within the genus *Timmia* have been well resolved through combined analyses of three DNA loci (*atp*B-*rbc*L spacer, *trn*L-*trn*F and 26S portion) (Budke and Goffinet 2006; Hedenäs 2011, 2021). To determine the phylogenetic position of the unknown *Timmia* species from the Sanjiangyuan Region, Qinghai Province, China, we included this specimen (voucher specimen: *W.-Z. Huang 20250827-06*) in the phylogenetic analysis. Additionally, one specimen of *T. bavarica* Hessl. (voucher specimen: *Zhang_MY20240807-44*) from China was also included. Both specimens were deposited in the Herbarium of Hangzhou Normal University (HTC). Two accessions, *Funaria hygrometrica* Hedw. and *Diphyscium foliosum* (Hedw.) D.Mohr, were selected as outgroups, while the remaining 44 accessions were obtained from GenBank (https://www.ncbi.nlm.nih.gov/). A list of taxa, including collection localities, vouchers, herbarium codes and GenBank accession numbers, is shown in Suppl. material 1.

### DNA extraction, sequencing, assembly and annotation

Sample preparation and DNA extraction followed protocols used in previous studies (Huang et al. 2019). High-quality genomic DNA from each sample was utilised for whole genome sequencing to obtain paired-end 150 bp raw reads on the Novaseq-SE50 platform according to the manufacturer’s procedures, accumulating about 5.3 Gb of sequences. Raw reads with a Phred score lower than 30 were removed, retaining high-quality sequences for nuclear DNA and complete circular organelle genome assembly using GetOrganelle v.1.7.7.1 (Jin et al. 2020). The assembly graph viewer Bandage (Wick et al. 2015) was employed to visualise the assemblies. Genomes were automatically annotated with CPGAVAS2 (Shi et al. 2019) and subsequently refined using Geneious v.11.0.3 (Kearse et al. 2012), using *Distichium capillaceum* (Hedw.) Bruch & Schimp. (accession number: OZ318515) as the reference plastome and then *atp*B-*rbc*L and *trn*L-*trn*F were extracted. The assembled nuclear data were aligned with published data using *Timmia megapolitana* Hedw. as a reference (26S accession number: DQ397105) in Geneious v.11.0.3 (Kearse et al. 2012) and then annotated and extracted.

### Phylogenetic analyses

All three sequences were aligned using MAFFT v.7.311 (Katoh and Standley 2013) and ambiguous alignment regions were trimmed using trimAl v.1.2 (Capella-Gutiérrez et al. 2009). Absent data were coded as missing. Maximum Likelihood (ML) analyses were performed in IQtree v.2.0.6 (Minh et al. 2020) with the sampling repeated 1000 times. The best-fitting substitution model (TN+F+I+G4 for 26S-partition, HKY+F+G4 for *atp*B-*rbc*L-partition and *trn*L-*trn*F-partition) was selected by ModelFinder (Chernomor et al. 2016; Kalyaanamoorthy et al. 2017) according to the Bayesian Information Criterion (BIC). For Bayesian Inference analyses, MrBayes 3.2.7 (Ronquist et al. 2012) was used to construct phylogenetic trees with 5,000,000 replicates, with trees sampled every 1000 generations. GTR+I+G is the best-fit model for 26S-partition, GTR+G for *atp*B-*rbc*L-partition and HKY+G for *trn*L-*trn*F-partition according to the Akaike Information Criterion (AICc). Markov Chain Monte Carlo (MCMC) was run independently twice with one cold and three hot chains. The posterior distribution of trees was summarised by 50% majority-rule consensus tree after discarding the first 25% of samples as burn-in.

### Morphological examination

Habit photographs and plant pictures were taken using a digital camera (Olympus TG6; Olympus, Tokyo, Japan) and a stereomicroscope equipped with an imaging system (Keyence VHX-6000; Keyence, Osaka, Japan). One specimen (*W.-Z. Huang 20250827-06*) was used for morphological study. Morphological observations were conducted with a Leica stereozoom microscope (Leica EZ4; Leica, Wetzlar, Germany) and an Olympus compound microscope (Olympus BX51; Olympus, Tokyo, Japan). Plant height, both tallest and shortest individuals, was measured using a ruler. Three plants were randomly selected and the lengths (from leaf insertion to apex) and widths (middle part of leaf sheaths) of all the leaves were measured. Microscopic images were captured with a digital camera (MOTICAM S6; Motic, Xiamen, China) mounted on the microscope and the size of the cells were measured using ImageJ software from these photos. SEM studies were performed on a Hitachi S-4800 Scanning Electron Microscope and the micrographs were taken from air-dried, gold-sputtered plants and spores.

## Results

**Figure 1.**
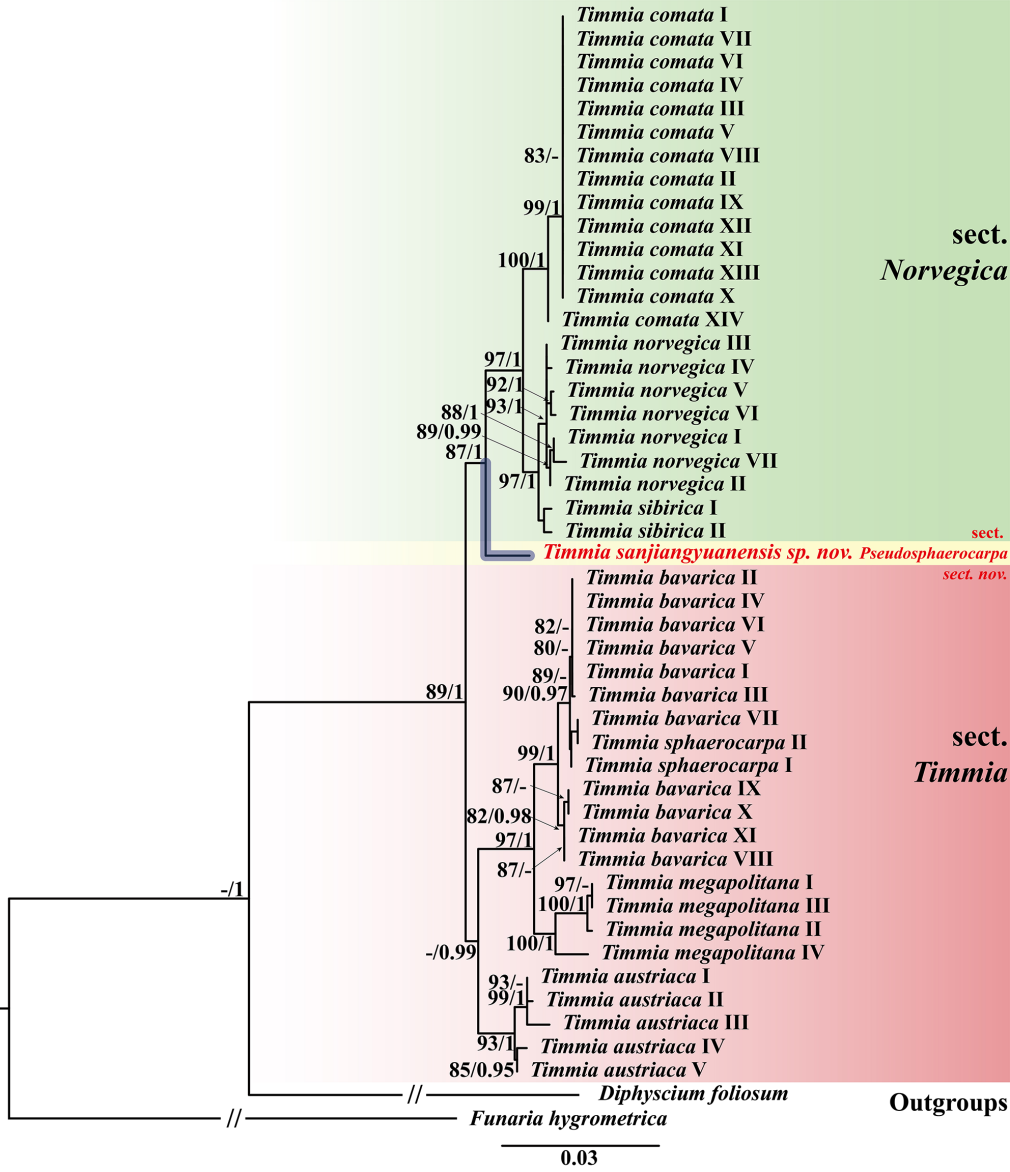
Phylogeny of *Timmia* species inferred from the combined dataset (*atp*B-*rbc*L, *trn*L-*trn*F and 26S). The topology derived from the best-scoring ML tree in IQtree is shown. ML bootstrap values BS ≥ 70 are shown on the left and Bayesian posterior probabilities values PP ≥ 0.90 on the right. The subject species are in red and indicated by arrows. ‘//’ means that the branch length shown in the representative picture is only half the actual length. Section division refers to Hedenäs (2021).

The aligned three-loci dataset included a total of 1,590 characters: *atp*B-*rbc*L spacer, 557 bp; *trn*L-*trn*F, 416 bp; and 26S, 617 bp. Amongst these 1,590 aligned nucleotides, 1,135 were constant sites, 290 were singleton sites and 165 were parsimony-informative sites. Both ML and BI analyses generated almost identical trees with strong support for most nodes. The ML topology tree with bootstrap (BS) and posterior probability (PP) values (BS_ML_ and PP_BI_, respectively) is shown in Fig. 1.

Within the phylogeny (Fig. 1), all *Timmia* species formed a well-supported clade (BS_ML_ = 89, PP_BI_ = 1). This clade resolved eight *Timmia* species into three distinct subclades: (1) sect. *Norvegica*, comprising *T. comata* Lindb. & Arnell, *T. norvegica* J.E. Zetterst. and *T. sibirica* Lindb. & Arnell (BS_ML_ = 97, PP_BI_ = 1); (2) a clade represented solely by the newly-sequenced specimen from the Sanjiangyuan Region, Qinghai Province, China; and (3) sect. *Timmia*, including *T. austriaca* Müll. Hal., *T. bavarica*, *T. megapolitana* and *T. sphaerocarpa* (PP_BI_ = 0.99). Notably, two accessions of *T. sphaerocarpa* were deeply nested within *T. bavarica* (BS_ML_ = 99, PP_BI_ = 1).

## Discussion

**Figure 2.**
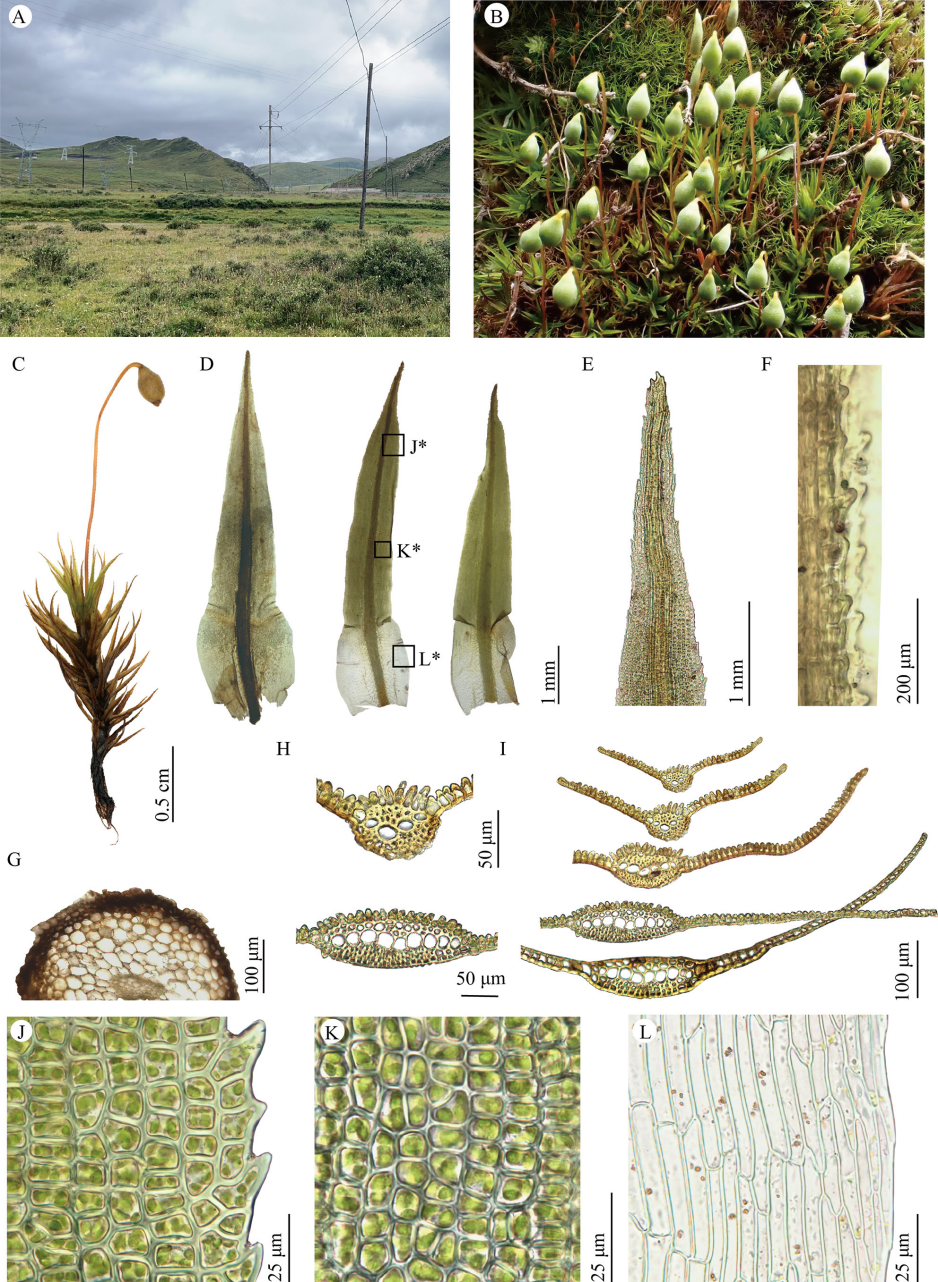
*Timmia sanjiangyuanensis* W.Z.Huang, X.Yin Ma & Y.Huan Wu. **A, B**. Habitat; **C**. Plant; **D**. Leaves; **E**. Leaf apex; **F**. Ventral surface of the costa; **G**. Transverse-section of stem; **H, I**. Transverse-sections of leaf; **J**. Upper marginal laminal cells of leaf; **K**. Middle laminal cells of leaf; **L** .basal laminal cells of leaf. All from *W.-Z. Huang 20250827-06* (holotype: HTC!).

**Figure 3.**
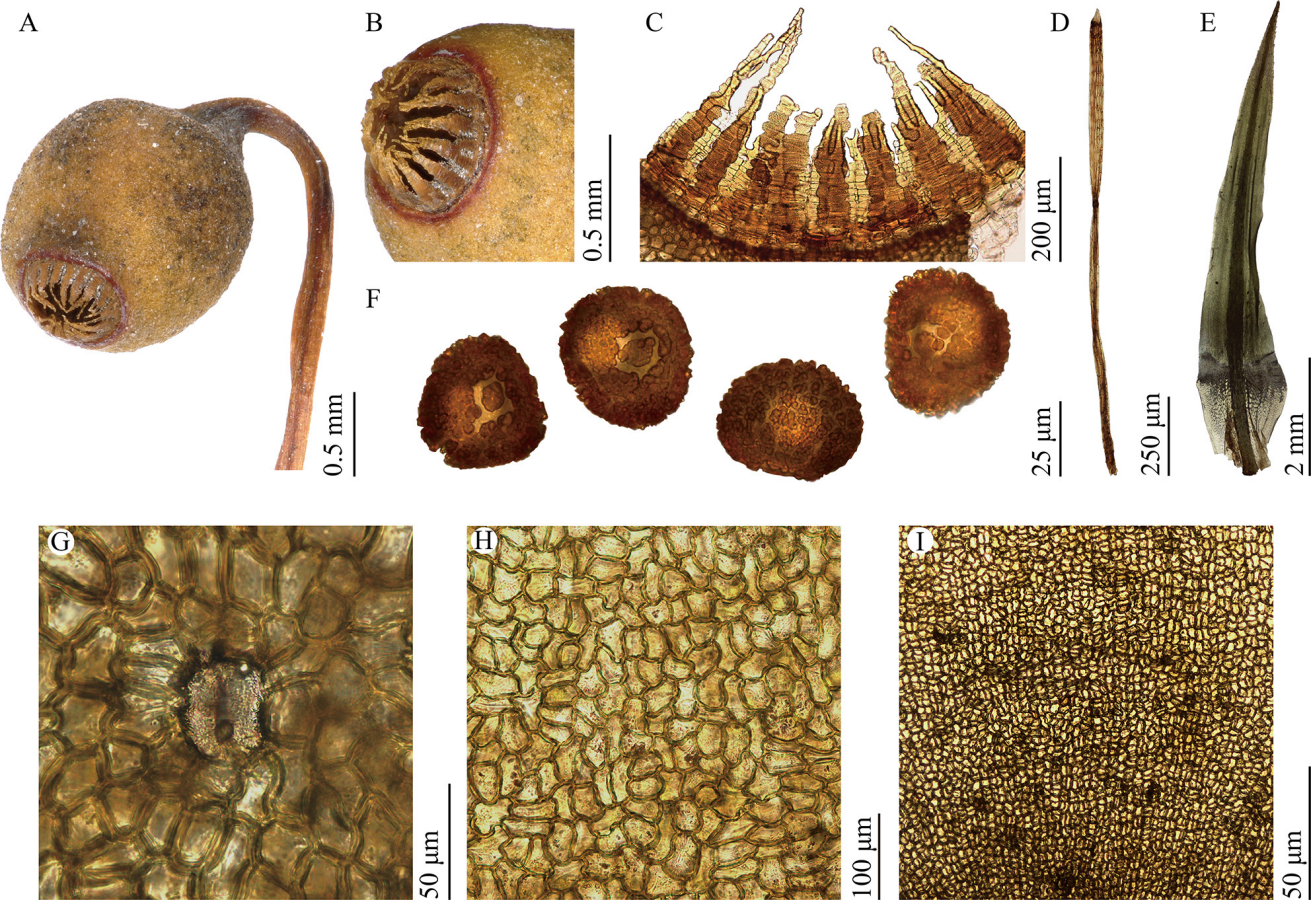
*Timmia sanjiangyuanensis* W.Z.Huang, X.Yin Ma & Y.Huan Wu. **A**. Capsule; **B, C**. Peristome; **D**. Antheridium; **E**. Perichaetial leaves; **F**. Spores; **G**. Stomata; **H**. Exothecial cell; **I**. Stomata at the base of the capsule. All from *W.-Z. Huang 20250827-06* (holotype: HTC!).

**Figure 4.**
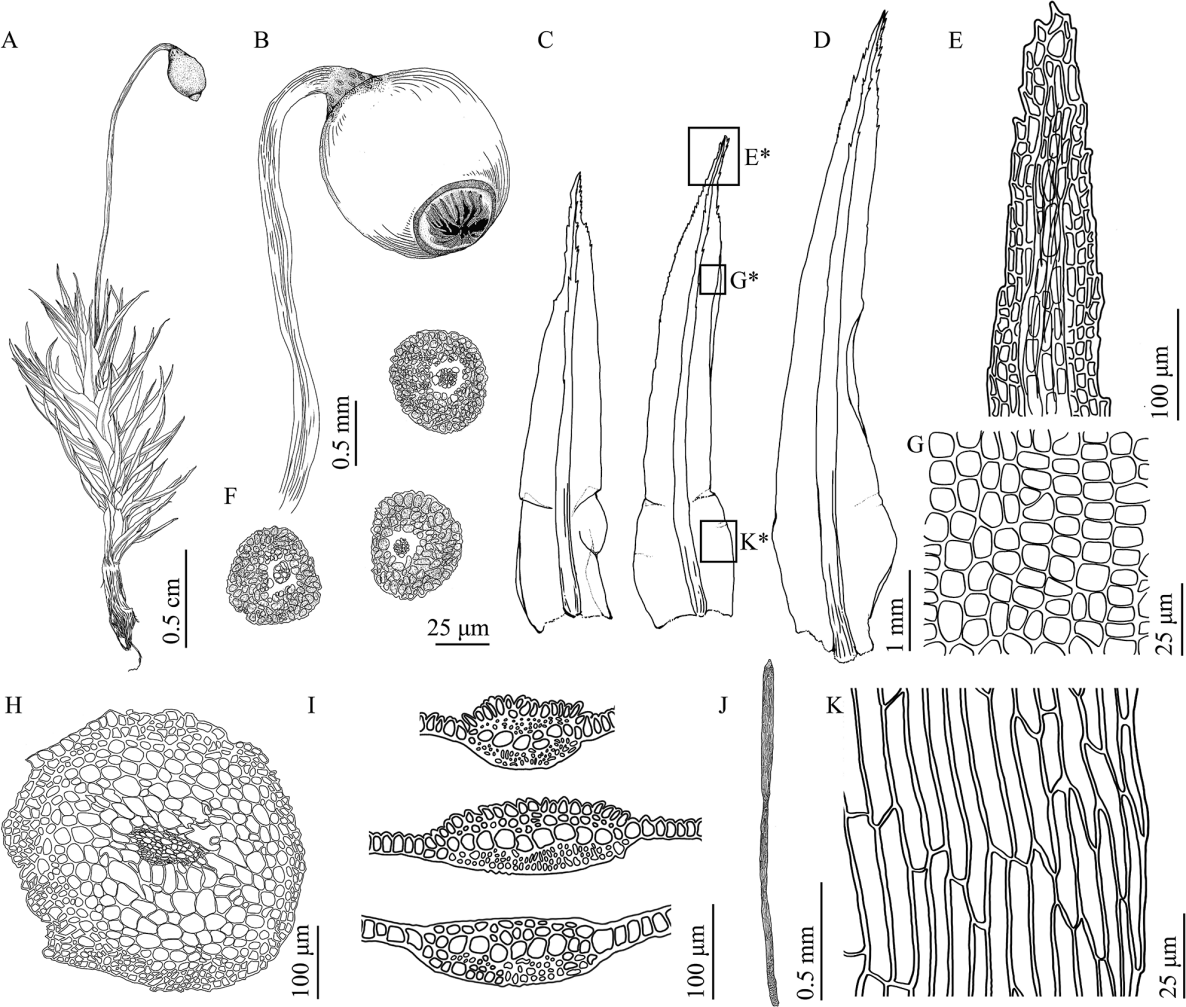
*Timmia sanjiangyuanensis* W.Z.Huang, X.Yin Ma & Y.Huan Wu. **A**. Plant; **B**. Capsule; **C**. Leaves; **D**. Perichaetial leaf; **E**. Leaf apex; **F**. Spores; **G**. Middle laminal cell of leaf; **H**. Transverse-section of stem; **I**. Transverse-sections of leaf; **J**. Antheridium; **K**. Basal laminal cells of leaf. All from *W.-Z. Huang 20250827-06* (holotype: HTC!).

**Figure 5.**
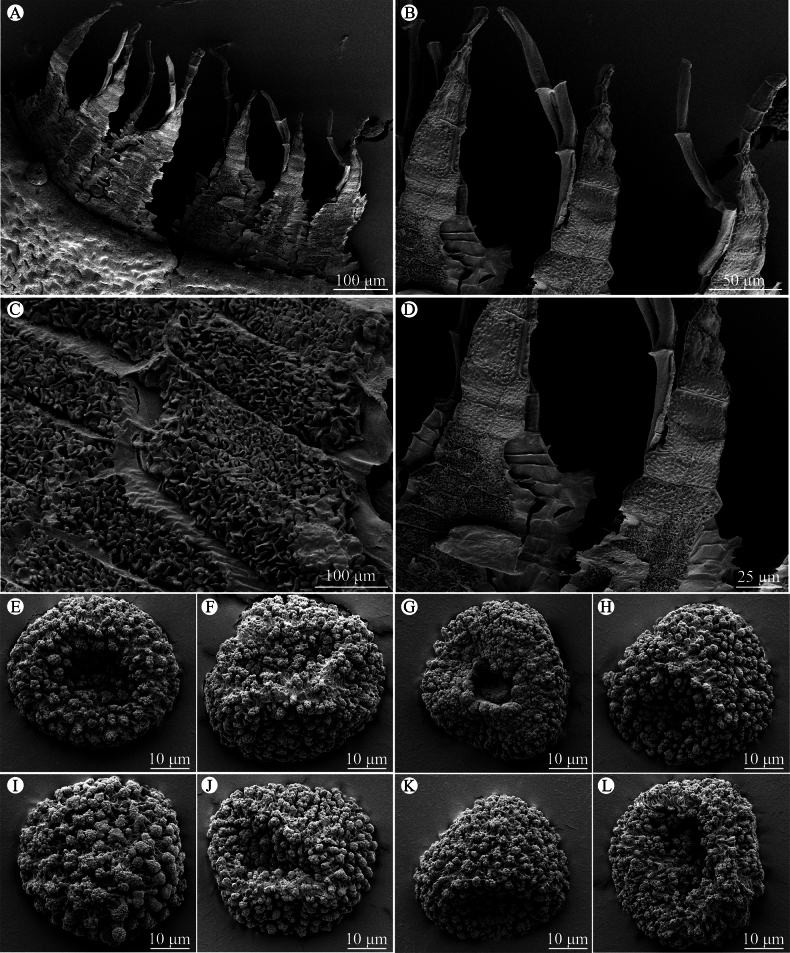
SEM of *Timmia sanjiangyuanensis* W.Z.Huang, X.Yin Ma & Y.Huan Wu. **A**. Outer surface of exostome; **B**. Detail of outer surface of exostome tooth and inner surface of endostome segment; **C, D**. Detail of outer surface of exostome tooth; **E–L**. Spores. All from *W.-Z. Huang 20250827-06* (holotype: HTC!).

The *Timmia* species from Qinghai Plateau closely resembles *T. sphaerocarpa* in its spherical capsules (Figs 2C, 3A, 4A, 4B; Jia and Liu (2006); Sun et al. (2013)). This distinctive feature separates them from all other known *Timmia* species, which possess linear-cylindrical capsules, such as *T. alataviensis* Müll.Hal., *T. austriaca*, *T. bavarica*, *T. comata*, *T. megapolitana*, *T. norvegica* and *T. sibirica* (Müller 1898; Brassard 1979, 1980, 1984, 2007; Jia and Liu 2006). Despite superficial similarity, these two species can be distinguished by the following characteristics: (1) stomata are distributed over the entire capsule in *T. sphaerocarpa* (Jia and Liu 2006), whereas, in this studied species, they are restricted to the capsule base (Figs 3I, 4B); (2) vegetative and perichaetial leaves exhibit a well-differentiated sheathing base in the studied species (Figs 2D, 3E, 4C, 4D), while those of *T. sphaerocarpa* are only slightly or not differentiated (Jia and Liu 2006); (3) limb laminal cells are strongly mamillose on the ventral surfaces in this studied species (Figs 3I, 4I), compared to the slightly mamillose cells in *T. sphaerocarpa* (Jia and Liu 2006); (4) antheridia are naked in the leaf axils of the studied species, but are enclosed by perigonial leaves in *T. sphaerocarpa* (Jia and Liu 2006); (5) exothecial cells are sinuose in the studied species (Fig. 3H), but non-sinuose in *T. sphaerocarpa* (Jia and Liu 2006) and (6) spores are significantly larger (36–46 μm in diameter; Figs 3F, 4F, 5E–L) in the studied species, whereas those of *T. sphaerocarpa* measure only 13.8–15.9 μm in diameter (Jia and Liu 2006). Therefore, this unknown species from the Qinghai Plateau does not belong to any currently known *Timmia* species. Both morphological and molecular data suggest that this moss from the Qinghai Plateau represents a species new to science, herein described as *Timmia sanjiangyuanensis* W.Z.Huang, X.Yin Ma & Y.Huan Wu.

The morphological similarity in capsule shape suggests a potential affinity between *Timmia sanjiangyuanensis* and sect. *Sphaerocarpa* (Jia and Liu 2006). Recently, sect. *Sphaerocarpa* was synonymised under sect. *Timmia* based on molecular data (Hedenäs 2021). Our phylogenetic analyses also support Hedenäs’s (2021) findings, showing that sect. *Sphaerocarpa* is deeply nested within sect. *Timmia* (Fig. 1). Therefore, we hypothesise that *T. sanjiangyuanensis* belongs to the sect. *Timmia*, as stated in the Introduction, based on morphological characteristic. However, our phylogenetic analyses indicate that *T. sanjiangyuanensis* does not form a clade with sect. *Timmia*, but is instead supported within sect. *Norvegica* (Fig. 1).

Notably, *Timmia sanjiangyuanensis* differs from species in sect. *Norvegica* in several key traits: (1) *T. sanjiangyuanensis* is autoicous, whereas all species in sect. *Norvegica* are dioicous (Crum 1967; Mastracci 1993; Tanaka and Tateishi 2002); (2) *T. sanjiangyuanensis* possesses a distinctly differentiated leaf sheath (Figs 2D, 3E, 4C, 4D), in contrast to the absent or only slightly differentiated and gradually widening sheath in sect. *Norvegica* (Brassard 1979, 2007; Jia and Liu 2006); (3) the leaf sheath cells are smooth in *T. sanjiangyuanensis* (Fig. 2L), whereas they are strongly mamillose on the dorsal surface of the lower leaves in section *Norvegica* (Mastracci 1993); (4) the capsules are pyriform to spherical in *T. sanjiangyuanensis* (Figs 2B, 2C, 3A), but liner-cylindrical in sect. *Norvegica* (Mastracci 1993; Tanaka and Tateishi 2002; Jia and Liu 2006); (5) the endostome cilia are non-appendiculate in *T. sanjiangyuanensis* (Fig. 4A, B, D), contrasting with the bluntly appendiculate cilia in section *Norvegica* (Crum 1967; Brassard 1979, 2007; Mastracci 1993; Budke and Goffinet 2006; Jia and Liu 2006; Li and He 2007); and, most importantly, (6) cells at leaf insertion are hyaline and fragile, with leaves readily breaking away from stem in sect. *Norvegica* (Brassard 1979, 2007), whereas these traits are absent in *T. sanjiangyuanensis* (Figs 2C, 4A). Given its unique phylogenetic position and suite of distinctive morphological characters, we hereby propose the establishment of a new section, sect. *Pseudosphaerocarpa*, to accommodate this remarkable species.

In China, Mitten (1859) recorded *Timmia austriaca* from Xizang, marking the first record of this genus in the country. Later, Müller (1898) documented the distribution of *T. alataviensis* and *T. megapolitana* in China. Subsequently, Brotherus (1929) recorded *T. bavarica* in Yunnan Province. Zhao (1991), Zhao and Han (1999) and Jia and Liu (2006) reported the distribution of *T. norvegica*, *T. comata* and *T. sphaerocarpa* in China, respectively. With the discovery of a new species from Qinghai Plateau, the number of *Timmia* species in China has increased to eight. To date, all known *Timmia* species have been recorded in China, except for *T. sibirica*. However, “Flora Bryophytorum Sinicorum” (Li 2006) and “Moss Flora of China” (Li and He 2007) include only five *Timmia* species. Therefore, providing an updated key to the *Timmia* species in China is essential and we present it herein.

### Key to *Timmia* species in China

(*T. alataviensis* was excluded since this species was poorly characterised and lacked illustrations when published (Müller 1898) and it was classified as an “insufficiently known” species by Crosby et al. (1999)).

**Table:** 

1	Cells> at leaf insertion hyaline and fragile, leaves readily breaking away from stem	**2**
–	Cells> at leaf insertion not hyaline and fragile, leaves not readily breaking away from stem	**3**
2	Dorsal surface of the costa generally mamillose; cells in the mid-limb region (8–) 9–14 μm wide	***T. norvegica***
–	Dorsal surface of the costa generally smooth or with a few teeth near the apex; cells in the mid-limb region 6–9 (10) μm wide	***T. comata***
3	Costa in transverse section lacking stereids in the leaf sheathing portion	***T. austriaca***
–	Costa in transverse section with stereids in the sheathing portion	**4**
4	Laminal cells in the upper part of leaf sheaths papillose (in dorsal view)	***T. megapolitana***
–	Laminal cells of leaf sheaths smooth	**5**
5	Upper laminal cells slightly mamillose on the ventral surface; stomata distributed over the entire capsule	***T. sphaerocarpa***
–	Upper laminal cells strongly mamillose on the ventral surface; stomata confined to the basal part of the capsule	**6**
6	Cells> in the mid-limb region 6–9 μm wide; capsule linear-cylindrical; spores 14–18 μm	***T. bavarica***
–	Cells> in the mid-limb region 10–16 μm wide; capsule pyriform or spherical; spores 36–46 μm	***T. sanjiangyuanensis***

### Taxonomy

#### Timmia section Pseudosphaerocarpa

Taxon classificationPlantaeTimmialesTimmiaceae

W.Z.Huang, X.Yin Ma & Y.Huan Wu sect. nov.

B793F800-A248-508B-81E4-ABA16F9DCBBE

##### Type.

*Timmia sanjiangyuanensis* W.Z.Huang, X.Yin Ma & Y.Huan Wu.

##### Description.

***Plants*** without deciduous leaves; ***leaf sheaths*** well-differentiated in vegetative and perichaetial leaves; ***antheridia*** naked; ***endostome*** cilia non-appendiculate; ***exothecial cells*** sinuose; autoicous; ***spores*** 36–46 μm in diameter.

#### Timmia sanjiangyuanensis

Taxon classificationPlantaeTimmialesTimmiaceae

W.Z.Huang, X.Yin Ma & Y.Huan Wu sp. nov.

5EB11655-1DFA-5199-99F0-A9DEF98AAFFB

Figs 2, 3, 4, 5

##### Diagnosis.

Similar to *Timmia sphaerocarpa*, but differing in having stomata restricted to the basal part of the capsule; vegetative and perichaetial leaves with a distinctly differentiated sheathing base; limb laminal cells with strong mamillose; antheridia naked in the leaf axils; sinuose exothecial cells and larger spores (36–46 μm in diameter).

##### Type.

China • Qinghai Province, Guoluo Zangzu Autonomous Prefecture, Sanjiangyuan Region, Gande County, Qingzhen Town, along G227 road, on soil, 34°5'48.90"N, 100°7'0.73"E, 4149 m a.s.l, 27 August 2025, *W.-Z. Huang 20250827-06* (holotype: HTC! [HTC0022010]; isotypes: HSNU!, KUN!).

##### Description.

***Plants*** robust, in loose tufts, brownish below, green to yellowish-green above. ***Stems*** simple, 1.5–2.4 cm, central strand present. ***Rhizoids*** red-brown. ***Leaves*** lanceolate, acuminate, 5.0–6.3 × 1.2–1.6 mm, with well-differentiated sheathing base. Leaf margins serrate in distal 1/3–1/2, entire below. ***Costa*** single, robust, subpercurrent, smooth on the dorsal surface, strongly mamillose on the ventral surface in the limb, smooth in the sheath; transverse-section of the costa with one row of guide cells, with two stereid bands in the limb, the ventral stereid band poorly developed or absent in the sheath. ***Cells of limb*** strongly mamillose on the ventral, smooth on the dorsal side, quadrate, irregularly polygonal or horizontal rectangular, 4.0–14.0 × 10.0–16.0 μm. ***Laminal cells*** of the sheath clearly differentiated, long-rectangular, hyaline, smooth, 23.5–84.0 × 2.5–8.5 μm, gradually longer and narrower towards the margin.

Autoicous. ***Antheridia*** naked in the leaf axils, 1.3–1.5 mm long. ***Perichaetial leaves*** lanceolate, widest just above the base, with a well-differentiated sheathing base, conspicuously larger than the vegetative leaves. ***Seta*** smooth, reddish, ca. 1.8 cm long. ***Capsule*** pyriform when wet, globose when dry and empty, ca. 1.4 mm in diameter, yellow-green when immature, yellow or brown and distinctly furrowed when mature. ***Stomata*** restricted to the basal part of the capsule. ***Exostome teeth*** 16, yellow at the base and pale yellow above, gradually tapering upwards; outer surface papillose and horizontally striate below, vertically barred and papillose above. ***Endostome segments*** 16, triangularly lanceolate, joined at base, inner surface of segments smooth, outer surface slightly granular above, not appendiculate. ***Operculum*** conical. ***Exothecial cells*** irregular, 14–34 × 16–32 μm, with thick and extremely sinuose walls throughout. ***Spores*** spherical, yellowish-brown, finely papillose, 36–46 μm in diameter.

##### Etymology.

The epithet “sanjiangyuanensis” refers to the Sanjiangyuan Region, where the new species was found.

##### Distribution and habitat.

*Timmia sanjiangyuanensis* is known only from Gande County in Sanjiangyuan Region, Qinghai Province, China, at an altitude of 4,149 m a.s.l, associated with *Distichium hagenii* Ryan ex Philib, *Encalypta ciliata* Hedw. and *Pohlia elongata* Hedw.

##### Notes.

*Timmia sanjiangyuanensis* is well characterised by the following features: (1) pyriform to spherical capsules with stomata confined to the basal part (Figs 2C, 3A, 3I, 4A, 4B); (2) exothecial cells irregular, extremely sinuose (Fig. 3H); (3) vegetative and perichaetial leaves lanceolate, with a well-differentiated sheathing base (Figs 2D, 3E, 4C, 4D); (4) antheridia naked in the leaf axils; (5) costa of the limb portion strongly papillose on the ventral surface, smooth on the dorsal surface and smooth on both dorsal and ventral sides in the sheath portion (Figs 2H, 2I, 4I); (6) laminal cells of the sheathing base differentiated, long-rectangular, hyaline, smooth (Figs 2L, 4K); and (7) spores large, finely papillose, 36–46 μm in diameter (Figs 3F, 4F, 5E–L).

## Supplementary Material

XML Treatment for Timmia section Pseudosphaerocarpa

XML Treatment for Timmia sanjiangyuanensis

## Additional information

### Artificial Intelligence (AI) use

The authors accept full responsibility for the content of the manuscript, including the disclosure of any use of AI.

No AI tools were used in the preparation of this manuscript.

### Author ORCIDs

X.-Y. Ma https://orcid.org/0000-0003-0876-4497

J.-Y. Zheng https://orcid.org/0009-0000-6222-8818

X.-R. Xia https://orcid.org/0009-0006-7708-7716

X.-T. Song https://orcid.org/0000-0002-8108-452X

Y.-K. Shi https://orcid.org/0009-0007-2231-5168

Y.-H. Wu https://orcid.org/0000-0002-8139-2194

W.-Z. Huang https://orcid.org/0000-0001-5871-5699

### Data availability

All of the data that support the findings of this study are available in the main text or Supplementary Information.

## Supplementary materials

Supplementary material 1 Voucher information and GenBank accession numbers Data type docx Explanation note **table S1**. Voucher information and GenBank accession numbers of the specimens analysed in this study. Newly-sequenced specimens are set in bold and the newly-sequenced data have been deposited in the GenBase in National Genomics Data Center. https://binary.pensoft.net/file/1675832This dataset is made available under the Open Database License (http://opendatacommons.org/licenses/odbl/1.0/). The Open Database License (ODbL) is a license agreement intended to allow users to freely share, modify, and use this Dataset while maintaining this same freedom for others, provided that the original source and author(s) are credited. Xin-Yin Ma, Jia-Yi Zheng, Xin-Rui Xia, Xiao-Tong Song, Ya-Kun Shi, Yu-Huan Wu, Wen-Zhuan Huang
