# Supplementary material for: Timmia sanjiangyuanensis (Timmiaceae, Bryophyta), a new species from the Qinghai Plateau, China
Source: PhytoKeys. 2026 Jun 10;276:35–49. doi: 10.3897/phytokeys.276.176402 (PMC13276498; doi:10.3897/phytokeys.276.176402)
Supplement: Supplementary material 1 — Voucher information and GenBank accession numbers [file phytokeys-276-035_article-176402__-s001.docx]

**Supplementary Table S1.** Voucher information and GenBank accession numbers of the specimens analysed in this study. Newly sequenced specimens are set in bold, and the newly sequenced data have been deposited in the GenBase (Bu et al. 2024) in National Genomics Data Center (CNCB-NGDC Members and Partners 2022).“—” means data missing.

| **Species** | **Locate** | **Vouchers (herbarium code)** | ***trn*L-*trn*F** | ***atp*B-*rbc*L** | **26S** |
| --- | --- | --- | --- | --- | --- |
| *Diphyscium foliosum* | USA | *Goffinet 4595* (CONN) | DQ397195 | DQ397161 | DQ397128 |
| *Funaria hygrometrica* | Chile | *Goffinet 5576* (CONN) | DQ397198 | DQ397164 | DQ397131 |
| *Timmia austriaca* I | Canada | *Goffinet, Vitt & Hastings 1284* (CONN) | DQ397165 | DQ397132 | DQ397099 |
| *T. austriaca* II | USA | *Hermann 28700* (NY) | DQ397167 | DQ397133 | DQ397101 |
| *T. austriaca* III | Sweden | *Frahm 11.VIII.1981* (NY) | DQ397169 | DQ397135 | DQ397103 |
| *T. austriaca* IV | Russia | *Hephrdebba 51* (NY) | DQ397168 | DQ397134 | DQ397102 |
| *T. austriaca* V | Norway | *H.Bednarek-Ochyra et al. (Br. Svalb. Exs. 75-1987)* (NY) | DQ397189 | DQ397155 | DQ397122 |
| *T. bavarica* I | Canada | *Schofield & McIntosh 74733* (NFLD) | DQ397170 | DQ397136 | DQ397104 |
| *T. bavarica* II | Spain | *Brugués 5.V.1980* (NFLD) | DQ397175 | DQ397141 | DQ397109 |
| *T. bavarica* III | Sweden | *Frahm 6.VIII.1981* (NY) | DQ397177 | DQ397143 | DQ397111 |
| *T. bavarica* IV | Russia | *Onipehenko 105/95* (NY) | DQ397178 | DQ397144 | DQ397112 |
| *T. bavarica* V | Kazakhstan | *Whittemore 3868* (NY) | DQ397179 | DQ397145 | DQ397113 |
| *T. bavarica* VI | Morocco | *Cano & Ros 16.VI.1997* (NY) | DQ397181 | DQ397147 | DQ397115 |
| *T. bavarica* VII | USA | *Brassard 13809* (NFLD) | DQ397176 | DQ397142 | DQ397110 |
| *T. bavarica* VIII | China | *Tan 95-1735* (NY) | DQ397180 | DQ397146 | DQ397114 |
| *T. bavarica* IX | China | *Jia 7938* (PE) | DQ778615 | —— | —— |
| *T. bavarica* X | China | *Jia 7942* (PE) | DQ778616 | —— | —— |
| ***T. bavarica* XI** | **China: Qinghai** | ***Zhang_MY20240807-44* (HTC)** |  |  |  |
| *T. megapolitana* I | Canada | *Ireland 20088* (NFLD) | DQ397171 | DQ397137 | DQ397105 |
| *T. megapolitana* II | Russia | *Ignatov 3.VIII.1988* (NY) | DQ397173 | DQ397139 | DQ397107 |
| *T. megapolitana* III | USA | *Budke 101* (CONN) | DQ397174 | DQ397140 | DQ397108 |
| *T. megapolitana* IV | Japan | *Tanaka 2210* (NY) | DQ397172 | DQ397138 | DQ397106 |
| *T. norvegica* I | Switzerland | *Vanderpoorten 4022* (LG) | DQ397184 | DQ397150 | DQ397117 |
| *T. norvegica* II | Britain | *Vanderpoorten 3090* (LG) | DQ397183 | DQ397149 | DQ397116 |
| *T. norvegica* III | Canada | *Ireland 21263* (NY) | DQ397185 | DQ397151 | DQ397118 |
| *T. norvegica* IV | USA | *Lewis 164* (NY) | DQ397186 | DQ397152 | DQ397119 |
| *T. norvegica* V | Russia | *O.Afonina (Bryoph. Ross. et Civit. Coll. Exs. 78-1995)* (NY) | DQ397187 | DQ397153 | DQ397120 |
| *T. norvegica* VI | Canada | *Hedderson 6819* (RND) | DQ397193 | DQ397159 | DQ397126 |
| *T. norvegica* VII | Russia | *Ignatov 22.VII.1991* (NY) | DQ397188 | DQ397156 | DQ397123 |
| ***T. sanjiangyuanensis*** | **China: Qinghai** | ***W.-Z. Huang 2025082706* (HTC, HSNU)** |  |  |  |
| *T. sibirica* I | Canada | *Brassard 4410* (NY) | DQ397191 | DQ397157 | DQ397124 |
| *T. sibirica* II | Russia | *Safronova 24.VII.1992* (NY) | DQ397192 | DQ397158 | DQ397125 |
| *T. sphaerocarpa* I | China | *Jia 8047* (PE) | DQ778613 | —— | —— |
| *T. sphaerocarpa* II | China | *Jia 8100b* (PE) | DQ778614 | —— | —— |
| *T. comata* I | Norway | *T.Hallingbäck 44089* (S) | JF342741 | JF342795 | JF342768 |
| *T. comata* II | Sweden | *T.Hallingbäck 4115* (S) | JF342739 | JF342793 | JF342766 |
| *T. comata* III | Norway | *T.Hallingback 41862* (S) | JF342738 | JF342792 | JF342765 |
| *T. comata* IV | Sweden | *N.Hakelier* (S) | JF342728 | JF342782 | JF342755 |
| *T. comata* V | Sweden | *L.Hedenäs J88-319* (S) | JF342727 | JF342781 | JF342754 |
| *T. comata* VI | Finland | *T.Ulvinen* (S) | JF342725 | JF342779 | JF342752 |
| *T. comata* VII | Norway | *L.Hedenäs (S)* | JF342722 | JF342776 | JF342749 |
| *T. comata* VIII | Norway | *L.Hedenäs*(S) | JF342721 | JF342775 | JF342748 |
| *T. comata* IX | Sweden | *T.Hallingbäck 38175 (S)* | JF342740 | JF342794 | JF342767 |
| *T. comata* X | Russia | *O.Y.Pisarenko (Br. Sib. Exs. 71)* (S) | JF342735 | JF342789 | JF342762 |
| *T. comata* XI | Canada | *R.J.Belland 2898 (Br. Exs. Terr.-Nov. Labr. 104) (S)* | JF342734 | JF342788 | JF342761 |
| *T. comata* XII | Sweden | *N.Hakelier (S)* | JF342729 | JF342783 | JF342756 |
| *T. comata* XIII | Norway | *L.Hedenäs(S)* | JF342726 | JF342780 | JF342753 |
| *T. comata* XIV | Sweden | *Hakelier 18.VIII.1984* (NY) | DQ397190 | DQ397154 | DQ397121 |

**References**

Bu C, Zheng X, Zhao X, Xu T, Bai X, Jia Y, Chen M, Hao L, Xiao J, Zhang Z, Zhao W, Tang B, Bao Y (2024) GenBase: A nucleotide sequence database. Genomics Proteomics Bioinformatics 22(3): qzae047. https://doi.org/10.1093/gpbjnl/qzae047

CNCB-NGDC Members and Partners (2022) Database resources of the National Genomics Data Center, China National Center for Bioinformation in 2022. Nucleic Acids Res 50(D1): D27–D38. https://doi.org/10.1093/nar/gkab951
